# Recent Advances in Neural Circuits for Taste Perception in Hunger

**DOI:** 10.3389/fncir.2021.609824

**Published:** 2021-02-02

**Authors:** Ou Fu, Yasuhiko Minokoshi, Ken-ichiro Nakajima

**Affiliations:** ^1^Division of Endocrinology and Metabolism, National Institute for Physiological Sciences, Aichi, Japan; ^2^Department of Physiological Sciences, School of Life Science, SOKENDAI (The Graduate University for Advanced Studies), Okazaki, Japan

**Keywords:** taste, hunger, satiety, neural circuit, appetitive and consummatory behaviors

## Abstract

Feeding is essential for survival and taste greatly influences our feeding behaviors. Palatable tastes such as sweet trigger feeding as a symbol of a calorie-rich diet containing sugar or proteins, while unpalatable tastes such as bitter terminate further consumption as a warning against ingestion of harmful substances. Therefore, taste is considered a criterion to distinguish whether food is edible. However, perception of taste is also modulated by physiological changes associated with internal states such as hunger or satiety. Empirically, during hunger state, humans find ordinary food more attractive and feel less aversion to food they usually dislike. Although functional magnetic resonance imaging studies performed in primates and in humans have indicated that some brain areas show state-dependent response to tastes, the mechanisms of how the brain senses tastes during different internal states are poorly understood. Recently, using newly developed molecular and genetic tools as well as *in vivo* imaging, researchers have identified many specific neuronal populations or neural circuits regulating feeding behaviors and taste perception process in the central nervous system. These studies could help us understand the interplay between homeostatic regulation of energy and taste perception to guide proper feeding behaviors.

## Introduction

Food intake is essential for survival and the neural processes mediating hunger information guide animals to initiate appetitive food seeking and subsequent consummatory feeding. Taste is important for animals to evaluate the value of food (Lindemann, 2001). It plays multiple roles in appetitive as well as in consummatory behaviors. Many brain areas show different taste responses in hunger and in satiety, indicating that taste perception is modulated by internal state. In this review, we will briefly introduce studies on taste perception in the peripheral and central nervous system, and then focus on the recent findings about the neural circuits related to the modulation of sweet or bitter taste in hunger and how they are involved in feeding.

## Taste Sensation From the Tongue to the Brain

**Figure 1:**
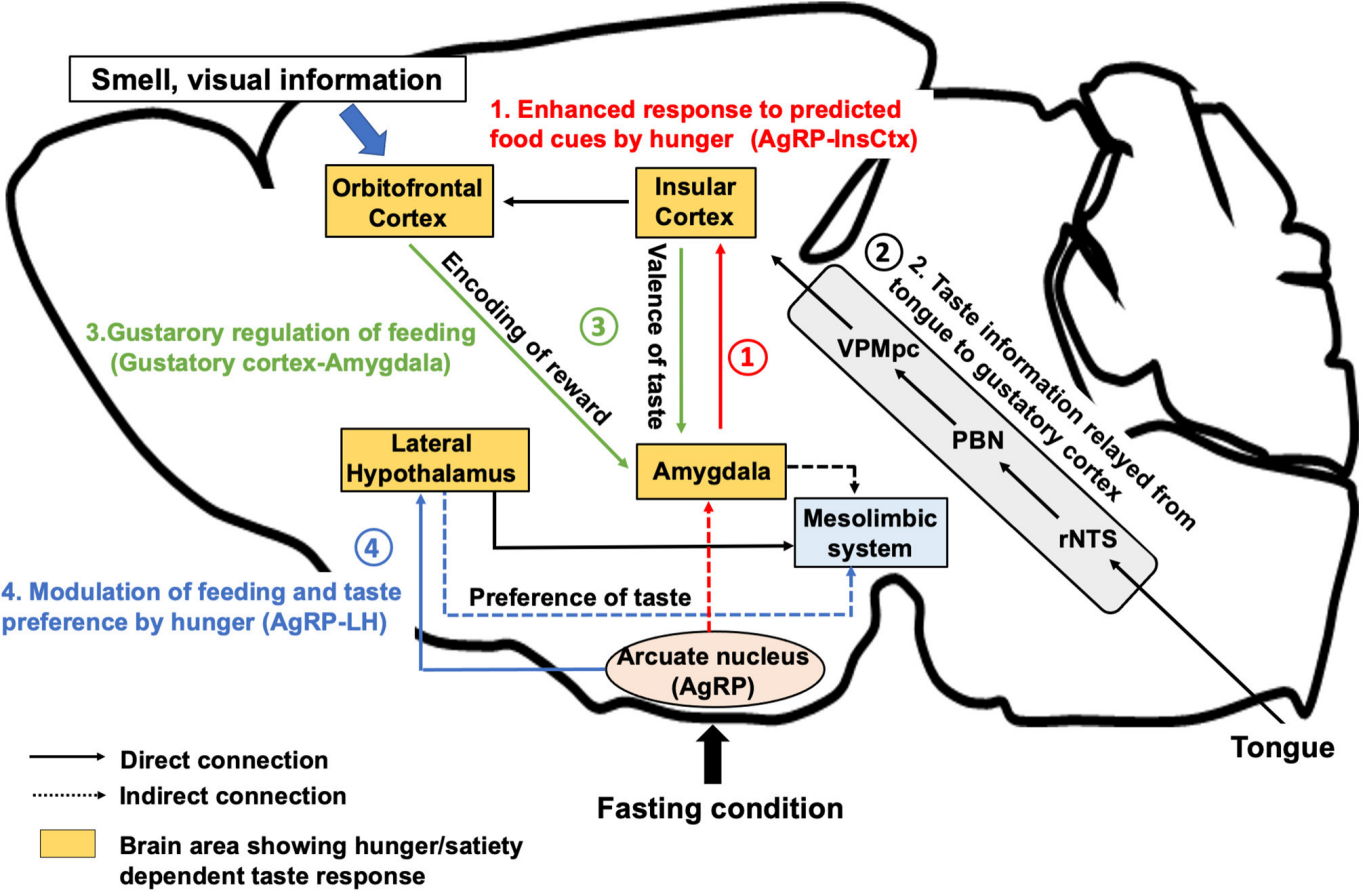
Neural regulation of taste perception in hunger state. AgRP neurons, which are firstly activated under hunger conditions, induce food seeking and consequent consummatory behaviors. **(1)** AgRP neurons inhibit the InsCtx via AgRP-PVT-BLA-InsCtx circuits to enhance the anticipatory response toward food cue in the appetitive feeding process. **(2)** After the start of eating, taste is detected by the tongue and perceived by the gustatory cortex (the InsCtx and the OFC) through rNTS, PBN, and VPMpc. **(3)** The InsCtx encodes the positive or negative valence of taste by interacting with the BLA or the central amygdala to continue or stop feeding. The OFC integrates taste and other sensory information as well as the reward value by projecting to the BLA. **(4)** Excitatory Vglut2-expressing neurons in the lateral hypothalamus also receives inhibitory inputs from AgRP neurons to induce feeding in hunger state. Vglut2 neurons in the LH project to the LS or to the LHb to modulate palatable or aversive taste preference, respectively. The aforementioned regions largely interact with the midbrain mesolimbic system to construct reward learning for the regulation of feeding behavior. BLA, the basolateral amygdala, CeA: the central nucleus of the amygdala, InsCtx: the insular cortex; LHb, the lateral habenula; LS, the lateral septum; VPMpc, the ventral posteromedial of the thalamus; OFC, the orbitofrontal cortex; PBN, the parabrachial nucleus; rNTS, the rostral part of the nucleus tractus solitarius.

Humans and many mammals are able to recognize five basic tastes: sweet, umami, bitter, sour and salty. Taste substances are detected by the taste buds on the tongue. They are onion-shaped structures composed of cells that express distinct types of taste receptors. For example, sweet substances are detected by a combination of T1R2 and T1R3 G-protein-coupled receptors, while bitter substances are detected by T2R receptors (Zhao et al., 2003; Meyerhof et al., 2005; Mueller et al., 2005). As shown in Figure 1, taste information is then sent to the brainstem via the taste ganglion to an area called the rostral part of the nucleus tractus solitarius (rNTS) (Beckstead and Norgren, 1979). In rodents, there is a relay from the rNTS to the parabrachial nuclei (PBN), which in turn project the information to the ventral posteromedial thalamic nucleus (VPMpc) (Beckstead et al., 1980; Pritchard et al., 1989). Eventually, the taste information is received at the primary gustatory cortex, which is also called the insular cortex (InsCtx) and at the secondary gustatory cortex, which is also called the orbitofrontal cortex (OFC) (Rolls et al., 1990). Although researchers have been aware of the gustatory neuronal pathways since many years, the molecular identity of individual gustatory neurons in the brain has rarely been discovered. Some recent studies have uncovered gustatory neurons that transmit specific taste information in mice. Pdyn-expressing neurons in the rNTS respond solely to sour taste and optogenetic activation of these neurons evokes aversion (Zhang et al., 2019). In a three-port apparatus, mice were provided access to the middle port to lick various solutions (water, bitter solution, or sour solution). They were trained to go to the left (bitter solution or water) or to the right port (sour solution) to report the taste identity. While mice reported correctly by going to the left port after licking water or bitter solution, Pdyn-neuron-activated mice went to the right port after licking water to report sour taste, suggesting that these neurons are required for sour taste recognition. Another study that focused on the PBN area indicated that Satb2-expressing neurons selectively respond to sweet taste and transmit the information to the VPMpc to evoke appetitive licking behaviors (Fu et al., 2019a). As fat besides sweeteners strongly induce appetitive feeding, it has raised the possibility of the taste of fat as the sixth taste modality. Although the oral perception of fatty acids has been previously thought to mainly rely on texture and olfaction (Rolls et al., 1999), the recent studies implied that several molecules such as CD36 and GPR120 play roles in fatty acid detection in peripheral taste systems. In CD36-positive taste cells in taste buds, Ca^2+^ concentration increased after application of long chain fatty acids (Gaillard et al., 2008). An increase in Ca^2+^ response to linoleic acid was observed in GPR120-expressing taste cells in mice (Ozdener et al., 2014). Moreover, electrophysiology recording of the mouse chorda tympani gustatory nerve indicate that GPR120 may play a role in distinguishing fatty acid taste from the other primary tastes (Yasumatsu et al., 2019).

Further studies should be conducted to clarify the molecular identity of other neurons associated with the gustatory response pathways to depict the complete taste-sensing network in the brain.

## The Role of Taste Perception in Feeding Behavior

Taste sensation has evolved to serve as a dominant controller of feeding behavior (Yarmolinsky et al., 2009). Feeding behaviors can be classified into homeostatic feeding to maintain body weight and metabolic function or hedonic feeding driven by sensory perception or pleasure (Berthoud, 2004; Rossi and Stuber, 2018). Notably, homeostatic and hedonic feeding systems are activated separately or simultaneously depending on various situations related to feeding (Castro et al., 2015). The activation pattern of these two systems may shift according to the taste of the food (palatable or aversive) and also according to the physiological state of the animal (hunger or satiety). Animals initiate feeding behavior in hunger state and terminate feeding when fed to satiety. This process is regulated by the homeostatic system to prevent excess caloric intake (Campos et al., 2016). However, palatable food could activate the mesolimbic reward system to induce food intake even under satiated condition. Hedonic feeding triggers appetitive behavior such as sugar craving, leading to overeating (Hajnal et al., 2004).

## Taste Perception and Internal State

Hungry and satiated animals exhibit different responses to attractive or potentially noxious taste substances. Neuroimaging studies in human subjects have shown that in hunger state, brain areas related to the reward system were more active after presentation of highly palatable food when compared with the same areas during satiety. This suggests that food becomes more palatable in hunger, which is described by an old saying “Hunger is the best spice” (Siep et al., 2009).

A previous study involving human taste evaluation task indicated that recognition thresholds for sucrose and salt were significantly lower during fasting state than during satiety (Zverev, 2004). Another study demonstrated that participants exhibited a significantly higher sensitivity to sweet, sour, and salty tastes during hunger state and a higher sensitivity to bitter taste during satiety (Hanci and Altun, 2016). These results indicate that internal state may directly influence the sensory perception of taste.

Some studies have also suggested that the palatability or the incentive value of taste is modulated by hunger. Hungry individuals show increased self-reporting of subjective feeling toward palatable taste (Rolls et al., 1983). Another study indicated that rats developed increased liking for sweet taste after starvation (Berridge, 1991). The authors evaluated the pleasantness of taste by using taste-reactivity facial expressions. Rats deprived of food for 48 h showed higher taste reactivity scores for sucrose–quinine mixture.

Several studies have reported that hormones and neuropeptides related to feeding may modulate peripheral taste sensitivity. Among various anorexic hormones, leptin is reported to selectively inhibit responses related to sweet taste in the taste receptor cells by biding to the functional leptin receptor Ob-Rb in the fungiform and circumvallate taste buds (Kawai et al., 2000; Shigemura et al., 2004; Yoshida et al., 2015). Glucagon-like peptide 1 (GLP-1) increases sensitivity to sweet taste via GLP-1 receptors located on the afferent nerve fibers adjacent to the taste buds (Shin et al., 2008), while cholecystokinin (CCK) on bitter taste via CCK-A receptors expressed in the taste receptor cells (Herness et al., 2002; Lu et al., 2003). Moreover, insulin seems to potentiate the response to salty taste via the epithelial sodium channels (ENaC) (Baquero and Gilbertson, 2011). Intranasal administration of insulin increased the sensitivity to sweet, bitter, salty, sour taste in human taste sensory tests (Rodriguez-Raecke et al., 2017). Neuropeptide Y, which is known as a orexigenic peptide, may influence the bitter taste sensitivity through NPY1R in the taste receptor cells (Zhao et al., 2005). Oxytocin is reported to decrease sweet sensitivity in mice via oxytocin receptors located in the taste buds (Sinclair et al., 2015). However, the central mechanism of taste modulation in hunger is still unclear.

## Brain Regions Showing Hunger-Dependent Taste Response

In the past, functional magnetic resonance imaging (fMRI) or electrophysiology studies have discovered brain areas including the OFC, the amygdala, the InsCtx, and the lateral hypothalamus (LH) that respond to taste cues in a hunger/satiety-dependent manner. Neuroimaging research in humans has shown that sated participants reported attenuated subjective pleasantness of a specific taste, suggesting that satiety has a negative effect on the taste perception (Rolls et al., 1983; Kringelbach et al., 2003). This decrease in pleasantness of taste showed a high correlation with a reduction in the activity of the OFC.

Electrophysiological investigations performed in primates revealed a large population of neurons in the OFC (Nakano et al., 1984; Yamamoto et al., 1984; Rolls et al., 1989), the amygdala (Nakano et al., 1986), and the LH (Burton et al., 1976) that responded to sucrose solution when the animal was hungry, but not when the animal was satiated. Importantly, simultaneous electrophysiological recording of single neurons from the OFC, the LH, and the amygdala was conducted in food-deprived rats with free access to sucrose solution (de Araujo et al., 2006). Similar to the results in primates, rats exhibited hunger-dependent response to tastes in these brain areas. However, due to the limitation of imaging resolution in the fMRI or electrophysiology studies, the precise role of these brain areas that showed state-dependent taste response remains largely elusive.

## Neural Circuits for Taste Perception and Correlated Feeding Behavior During Hunger

Under fasting condition, animals need to seek food, to decide eating or rejecting the food by savoring the taste, and to consume the food for survival. It is suggested that distinct neural circuits related to appetite, taste perception, and reward work together to evoke appetitive behavior and regulate the subsequent consummatory behavior (Rolls, 2005; Ferrario et al., 2016).

**Figure 2:**
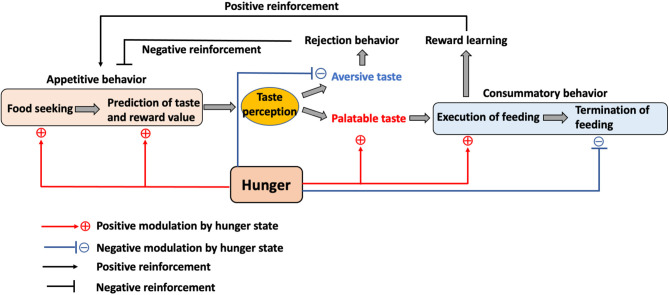
The role of the internal state and taste in feeding. Hunger motivates animals to initiate food seeking and to promote the taste and reward value prediction in the appetitive feeding process. While palatable taste leads to food consumption, aversive taste mediates rejection. This process also contributes to the positive or negative reinforcement toward food. Hunger enhances the preference toward palatable taste while reducing the aversion toward aversive taste to promote consummatory feeding and also inhibits the termination of feeding until satiety.

To understand the role of the gustatory system during hunger state, the feeding behavior can be classified into three phases: (1) motivational and anticipatory process before the start of feeding (appetitive behavior), (2) taste perception and execution of feeding (consummatory behavior), and (3) termination of feeding (Figure 2). In recent years, newly developed neural manipulation methods and *in vivo* calcium imaging have allowed the study of neuronal functions related to the gustatory system and feeding regulation using high time resolution.

## Motivational and Anticipatory Process Before the Start of Feeding

Hunger promotes motivation for appetitive behaviors to seek food and evokes feeding behaviors (Sternson et al., 2013). The hypothalamus has been considered the feeding center. It contains a variety of neuronal cell types associated with maintaining energy homeostasis. Among them, neurons that specifically express the Agouti-related protein (AgRP) within the arcuate nucleus of the hypothalamus have been identified as hunger neurons. Ablation of AgRP neurons in adult mice leads to aphagia (Wu et al., 2009). Conversely, utilization of optogenetic and chemogenetic techniques for rapid activation of cell-type-specific neurons showed that AgRP neurons are sufficient to evoke feeding behavior in fed mice within minutes and the amount of food intake is similar to that observed in overnight fasted mice (Krashes et al., 2011; Betley et al., 2013). Interestingly, GCaMP-based calcium imaging of AgRP neurons using fiber photometry has indicated that the activity of these neurons starts to decease even before a bite of the food (Chen et al., 2015), suggesting that AgRP neurons are involved in the anticipatory process to predict the caloric consumption. This anticipatory response for food sensory cues may help the transition process from seeking food to subsequent feeding behaviors.

Human fMRI studies have shown that the response of the InsCtx or the OFC to sweet solutions changes in a hunger-dependent manner (Haase et al., 2009). Taste solutions were orally delivered to hungry or satiated participants. Blood oxygenation level-dependent signal change measured by fMRI showed significant differences in the activation for sucrose, caffeine, saccharin, and citric acid in the OFC and in the InsCtx between hunger and satiety. Recent studies (Livneh et al., 2017, 2020) have demonstrated that a specific pathway from the hunger-promoting AgRP neurons to the InsCtx via the paraventricular thalamus (PVT) and the basolateral amygdala (BLA) is involved in hunger-dependent enhancement of food cue responses in mice (Figure 1-1). The authors applied a visual discrimination task wherein mice were trained to lick after different learned visual cues related to the delivery of a sweet solution (Ensure), a bitter solution (quinine), or water. GCaMP-based single-cell calcium imaging of the InsCtx neurons in behaving mice showed that a large population of InsCtx neurons responded to the visual food cue, licking, or sweet taste. The authors used quinine for the training of visual discrimination task to allow the mice to learn the negative food cue. However, during actual imaging, only Ensure was delivered to the mice. Interestingly, a large proportion of neurons related to food cue response were strongly activated when the mice licked the sweet solution. Imaging the same InsCtx neurons during hunger and satiety revealed that the neurons that were responsive to visual cues during hunger were abolished during satiety. Another human fMRI study suggested that seeing pictures of palatable food could activate the InsCtx (Simmons et al., 2005). These evidences suggest that the InsCtx represents food prediction and interoceptive consequences of upcoming consumption. Thus, this hypothalamus-cortical connection shows the possibility to enhance the palatability of food during hunger state.

A human fMRI study has shown that the OFC and the amygdala encode predictive reward value (Gottfried et al., 2003). *In vivo* calcium imaging study of the OFC neurons (Jennings et al., 2019), which are the direct projection targets of the InsCtx, demonstrated significant excitatory responses during caloric-reward licking in starved mice. Pairing each caloric-reward delivery with optogenetic stimulation of feeding-responsive cells significantly increased licking. Interestingly, stimulation of feeding-responsive cells does not increase licking of non-caloric sweetener saccharin, suggesting that OFC neurons are responsive to caloric reward content rather than to taste reward. It is also reported (Malvaez et al., 2019) that BLA-projecting OFC neurons encode the state-dependent incentive value of palatable food reward. Glutamate receptor activity in the BLA is necessary for reward value encoding and retrieval. Projections from the lateral OFC to the BLA are necessary and sufficient for encoding the positive value of a reward. On the other hand, projections from the medial OFC to the BLA are necessary and sufficient for retrieving this value from memory. A reinforcer devaluation paradigm study has suggested that the OFC and the BLA play different roles in mediating normal goal-directed performance (Pickens et al., 2003). The BLA seems critical to forming representations that link cues to the incentive properties of outcomes, but the OFC possibly contributes to maintaining these representations in memory and updating them with new information.

These results imply that the InsCtx and the OFC are not only responsive to taste identities but also play a role in the state-dependent reward prediction before feeding. While the InsCtx and the OFC are possibly involved in the prediction of taste as well as food reward, the OFC contains specific neuronal population that contributes to the prediction of the caloric content.

## Taste Perception During Feeding

Food cues such as visual information and smell allow animals to predict the gustatory identity as well as the reward value of the food (Rolls, 2015). The decision to execute the actual oral feeding action depends on the identity of taste. Gustatory pathways from the brainstem to the cortical areas are important to generate the perception of taste (Figure 1-2). Oral exposure to food with normal taste maintains the feeding action, while palatable tastes trigger positive feeling and increase the frequency of mastication. However, aversive tastes transmit negative feeling and lead to acute termination of feeding.

A recent study (Wang et al., 2018) demonstrated that the innervation between the InsCtx and the amygdala helps transmit taste valences (Figure 1-3). Gustatory cortices for sweet taste and bitter taste are located in the anterior and the posterior part of the InsCtx. They innervate distinct subnuclei in the amygdala (the BLA for sweet and the central nucleus of the amygdala [CeA] for bitter). Optogenetic activation of BLA-projecting InsCtx neurons increases water-licking behavior by evoking “virtual” sweet taste sensing, while activation of CeA-projecting InsCtx neurons decreases licking toward water by mimicking the bitter taste. Another study using a tastant (sucrose/quinine)-reinforced “go/no-go” task showed that specific inhibition of neurotransmitter release from the lateral CeA (CeL)-projecting InsCtx neurons prevented mice from acquiring the “no-go” responses for quinine and impaired the “go” responses for sucrose in the “go/no-go task,” indicating that the InsCtx-CeL circuit is important for the establishment of behavioral response to cues predicting appetitive or aversive tastants (Schiff et al., 2018). These results suggest that the InsCtx contains neurons that encode the innate taste valence as well as the learned taste cue prediction to evoke appetitive or rejection behaviors. However, it is unclear whether neurons related to gustatory response in the InsCtx are distinct from or overlap with the cue-predicting neurons in the InsCtx.

Considering the evidence of the connection between the amygdala and the mesolimbic reward system, the amygdala may function as a relay point to transmit the taste valence and the reward property (InsCtx-amygdala and OFC-amygdala) to trigger feeding behaviors.

## Taste and Internal State Modulation for the Termination of Feeding

During energy deficiency, animals require calories to maintain the body weight and the metabolic functions. They continue consummatory feeding from hunger state until satiety to meet the caloric need. The LH is an essential neuroanatomical region for appetitive as well as for consummatory behaviors (Nieh et al., 2015). The LH contains many distinct types of neurons and activation of GABAergic (Vgat-expressing) neurons in the LH enhances both appetitive and consummatory behaviors. Calcium imaging in freely behaving mice has indicated that individual Vgat^LH^ neurons preferentially encode aspects of appetitive or consummatory behaviors (Jennings et al., 2015). In contrast, glutamatergic Vglut2 neurons, which are also abundant in the LH, play an opposite role in regulating the feeding behaviors. Optogenetic stimulation of Vglut2^LH^ neurons not only decreases appetite in hungry mice but also produces an aversion to locations linked with the stimulation of these cells (Stamatakis et al., 2016). *In vivo* calcium imaging study on Vglut2^LH^ neurons demonstrated a greater response during sucrose delivery in satiety when compared with the response in hunger state (Rossi et al., 2019). Although these results are opposite to the results of fMRI or electrophysiology studies of LH showing greater response in hunger than in satiety, it could be suggested that distinct neuronal populations exist in the LH and Vglut2 neurons specifically encode satiety and serve as a brake to terminate the feeding action.

An electrophysiological study (Li et al., 2013) indicated that the LH contains two distinct populations of neurons responding to appetitive and aversive tastes. A recent study revealed that physiological hunger affects preferences to appetitive as well as aversive tastes in a mouse model. These effects are induced by LH-projecting AgRP neurons (Fu et al., 2019b). In a brief-access taste test, chemogenetic and optogenetic activation of AgRP neurons in fed animals increased the number of licks for a solution containing a relatively low concentration of sucrose. In contrast, elevation in the number of licks for a bitter (denatonium) solution was also observed after optogenetic activation of AgRP neurons. Furthermore, chemogenetic inhibition of Vglut2^LH^ neurons, but not of Vgat^LH^ neurons recapitulated the hunger-induced taste modification. Two distinct neuronal pathways from Vglut2^LH^ neurons to the lateral septum or to the lateral habenula (LHb) contribute to the modulation of appetitive and aversive taste, respectively. The LS and the LHb are parts of mesolimbic reward system, indicating that the LH may modulate the reward value of the taste (Figure 1-4). While Vglut2^LH^ neurons that encode satiety serve as a brake to terminate consummatory behavior during hunger state (Stamatakis et al., 2016; Rossi et al., 2019), these neurons receive inhibitory inputs from the AgRP neurons. Enhancement of preference to sweet tastants and tolerance to bitter tastants through AgRP-dependent inhibition of Vglut2^LH^ neurons could promote consummatory behavior to meet the emergent caloric need.

Notably, the OFC is connected both directly and indirectly with the LH (Öngür and Price, 2000; Reppucci and Petrovich, 2016), suggesting that the OFC may pass integrated sensory information into the LH, evoking motivation for palatable taste. Furthermore, the LH interacts with the mesolimbic system to induce reward learning for positive or negative reinforcement and to modulate the taste preference to trigger or to prevent future food consumption.

## Conclusion

Feeding behaviors are regulated by homeostatic and hedonic feeding systems. Taste perception from the brainstem to the gustatory cortex serves as a regulator and driving factor for feeding by interacting with the hypothalamus and with the mesolimbic reward system. The brain integrates sensory information and interoception to guide proper feeding behaviors for survival. Understanding the mechanism by which the brain senses taste and reward modalities in different physiological states may be beneficial in therapeutic treatment of overeating in the future.

## Author Contributions

All authors listed have made a substantial, direct and intellectual contribution to the work, and approved it for publication.

## Conflict of Interest

The authors declare that the research was conducted in the absence of any commercial or financial relationships that could be construed as a potential conflict of interest.
